# Cannabis use disorder is associated with shorter length of stay and increased home discharge after primary total joint arthroplasty: a propensity-weighted analysis

**DOI:** 10.1186/s42836-023-00164-9

**Published:** 2023-02-27

**Authors:** Dalia Rahmon, Leo Zalikha, Matthew Mazur, Inaya Hajj Hussein, Mouhanad M. El-Othmani

**Affiliations:** 1 Department of Foundational Medical Studies, Oakland University William Beaumont School of Medicine, Rochester, MI 48309 USA; 2 Department of Orthopaedic Surgery and Sports Medicine, Detroit Medical Center, Detroit, MI 48201 USA; 3 Department of Orthopaedic Surgery, Columbia University Medical Center, New York, NY USA

**Keywords:** Cannabis use disorder, Total joint arthroplasty, Total hip arthroplasty, Total knee arthroplasty, Epidemiology, Demographics, Complications, Outcomes

## Abstract

**Introduction:**

Increased legalization of cannabis use across the United States has been correlated with increased cannabis use in the clinical setting. However, little is known regarding the characteristics and postoperative outcomes after primary joint arthroplasty (TJA) for patients with cannabis use disorder (CUD).

**Methods:**

This retrospective cohort study used data from the National Inpatient Sample of patients undergoing primary TJA between 2006 to 2015. Patients were grouped based on presence of concomitant CUD. Patient demographic characteristics and outcome data between groups were analyzed. Propensity score methodology was used to compare immediate in-hospital complications and economic outcomes.

**Results:**

A total of 8,740,798 TJAs were included. The prevalence of CUD increased nearly five-fold from 0.05% to 0.26% during this time (*P* < 0.0001). CUD patients were significantly younger, more likely to be male, most frequently of non-Hispanic Black race, and had higher rates of Medicaid insurance. Patients with CUD had a significantly shorter length of hospital stay (3.04 *vs.* 3.24 days, *P* = 0.0297), while incurring significantly higher daily ($22,614 *vs.* $17,955, *P* < 0.0001) and total charges during admission ($58,507 *vs.* $50,924, *P* < 0.0001), compared to patients without CUD. When compared with the control group, CUD was associated with significantly greater odds of home discharge (odds ratio (OR): 1.45, *P* = 0.0007), and significantly lower odds of rehab discharge (OR: 0.70, *P* = 0.0013). There were no differences in overall complication profile or in the vast majority of individual in-hospital complications between groups.

**Conclusion:**

While CUD is correlated to shorter length of stay and increased home discharge after TJA, it does not show a strong effect on complications in an inpatient postoperative setting. It is important for clinicians to appreciate the demographic profile and expected clinical and economic outcomes for patients with CUD undergoing TJA, particularly in the context of evolving laws surrounding cannabis use.

## Introduction

Widespread legalization and decriminalization of cannabis across the United States has been associated with a considerable rise in self-reported cannabis use amongst surgical patients, including those undergoing total joint arthroplasty [1, 2]. While cannabis is primarily used for recreational purposes, cannabinoid metabolites have shown analgesic and anti-inflammatory properties and have thus been proposed as an alternative to opioids in the management of acute and chronic pain [2–5]. While cannabis use may conceivably be beneficial in the postoperative setting, cannabis use disorder (CUD), defined in part as a problematic pattern of cannabis use leading to clinically significant impairment or distress, has been correlated with increased postoperative pain and opioid use following orthopedic surgical procedures [6–12]. It remains unclear what potential utilization and implication the cannabis use has on postoperative clinical and economic outcomes of patients undergoing major elective surgery, such as total joint arthroplasty (TJA) [2, 3, 10, 11, 13]. The relevant literature on this topic has yielded conflicting results, while being complicated by a patient population that is constantly evolving in the context of social and legal changes leading to increased cannabis use [14].

Total hip arthroplasty (THA) and total knee arthroplasty (TKA), collectively referred to as total joint arthroplasty (TJA), are among the most frequently performed surgeries in the United States [15]. Literature evaluating demographic and outcome trends for CUD patients undergoing TJA is altogether lacking. The rise in self-reported cannabis use paralleling continuously increasing rates of TJA has made it extremely relevant and worthwhile for healthcare professionals to better understand this population. Furthermore, given increased pressures in national healthcare systems aimed at improving quality and minimizing variability in outcomes, it is crucial to identify potential implications of CUD on in-hospital postoperative outcomes after TJA. Investigating these topics will help address current gaps in the literature while providing insight into the most appropriate clinical care and perioperative interventions for this patient population.

The main objectives of this study were to highlight trends in cannabis use disorder among TJA patients, evaluate epidemiological and demographic characteristics of TJA patients with and without CUD, and compare inpatient postoperative clinical and economic outcomes among patients with and without CUD.

## Materials and methods

Discharge data from 2006 to 2015 were collected from the National Inpatient Sample (NIS) database and retrospectively analyzed in this study. The NIS was developed for the Healthcare Cost and Utilization Project (HCUP) and is the largest publicly available all-payer inpatient hospital database in the United States. The NIS contains data from over seven million hospital stays, representing a 20% stratified sample of discharges from community hospitals across the US. The *International Classification of Disease, the Ninth Revision, Clinical Modification (ICD-9-CM)* coding system was used during this study period. Institutional review board exemption was approved for this study.

TJA patients were considered to be those undergoing either a primary THA or TKA. Patients older than 40 years who underwent a primary THA (*ICD-9-CM*, code 81.51) or primary TKA (81.54) were included in this study. The TJA population was then divided into two groups: (1) patients with cannabis use disorder (CUD), and (2) patients without CUD (referred to as the control group). Cannabis use disorder (CUD) was identified using *ICD-9-CM* codes 304.30, 304.31, 304.32, and 305.2. Exclusion criteria involved patients below the age of 40 years old, those undergoing revision procedures, and patient codes containing the specifier "in remission". The term “any complication” was used to refer to any postoperative cardiac, gastrointestinal (GI), genitourinary (GU), hematoma/seroma, wound dehiscence, infection, deep vein thrombosis (DVT), pulmonary embolism (PE), or anemia complications. Patient epidemiological and demographic characteristics, comorbidities, hospital length of stay, discharge disposition, immediate in-hospital complications, and economic outcomes of cohorts with and without CUD were then comparatively analyzed.

Appropriate NIS trend weights were employed for analysis [16]. Discharge characteristics were documented, and analysis was performed according to recommendations from the Agency for Healthcare Research and Quality (AHRQ) [17, 18]. Differences in patient characteristics, such as age, gender, and comorbidities, were analyzed using *t*-tests and univariate logistic regressions, and further elucidated using propensity score matching through a combination of the Elixhauser Comorbidity Index and Inverse Probability of Treatment Weights (IPTW) [19–21]. Statistical significance was defined at *P* < 0.05. All data analyses were performed using SAS 9.4 (SAS Institute Inc., Cary, NC, USA) and Stata 13 (StataCorp, LLC, College Station, TX, USA).

## Results

### Trends in CUD by year

An estimated total of 8,740,798 TJAs were examined from 2006 to the third quarter of 2015. This total consisted of 2,838,741 THA and 5,902,057 TKA procedures. There was an approximately five-fold increase in the prevalence of CUD during this period, from 0.05% in 2006 to 0.26% in 2015 (*P* < 0.0001) (Table 1).

**Table 1:** Trend in cannabis use disorder rate by year in patients undergoing total joint arthroplasty

Year	Rate (95% CI)	***P***-Value
2006	0.05% (0.03%, 0.07%)	< 0.0001
2007	0.04% (0.03%, 0.05%)	
2008	0.07% (0.05%, 0.08%)	
2009	0.07% (0.05%, 0.09%)	
2010	0.07% (0.06%, 0.09%)	
2011	0.13% (0.10%, 0.17%)	
2012	0.13% (0.11%, 0.14%)	
2013	0.14% (0.12%, 0.16%)	
2014	0.20% (0.18%, 0.23%)	
2015	0.26% (0.23%, 0.29%)	

### Demographic and hospital factors

Significant differences in several demographic and hospital factors between CUD and non-CUD patients undergoing TJA were identified (Table 2). These differences were observed for all variables besides “Elective Admission” and “Bedsize of Hospital”. Specifically, patients with CUD were significantly younger, more often of male gender, more frequently had Medicaid insurance with lower rates of Medicare insurance, were more likely to be non-Hispanic Black, and were less likely to be non-Hispanic White when compared to the non-CUD cohort. Patients with CUD were seen most frequently in urban teaching hospitals and in Western regions of the United States.

**Table 2:** Demographic and hospital factors, stratified by CUD *vs*. no CUD

	CUD (***n*** = 10,450)	No CUD (***n*** = 8,728,240)	***P***-Value
Age (Years)			
Mean (Standard Error)	55.35 (0.18)	66.21 (0.04)	< 0.0001
Biological Sex of Patient			
Male	6,778 (64.86%)	3,429,533 (39.29%)	< 0.0001
Female	3,672 (35.14%)	5,298,707 (60.71%)	
Expected Primary Payor			
Medicare	3,182 (30.45%)	4,780,850 (54.77%)	< 0.0001
Medicaid	2,363 (22.62%)	268,762 (3.08%)	
Private	4,033 (38.60%)	3,346,741 (38.34%)	
Other	872 (8.34%)	331,885 (3.81%)	
Race of Patient			
Non-Hispanic White	6,430 (61.54%)	6,320,578 (72.42%)	< 0.0001
Non-Hispanic Black	2,320 (22.20%)	547,050 (6.27%)	
Hispanic	350 (3.35%)	349,886 (4.01%)	
Other Races	1,350 (12.92%)	1,510,725 (17.31%)	
Bedsize of Hospital			
Small	1,848 (17.68%)	1,708,714 (19.58%)	0.0733
Medium	3,065 (29.33%)	2,298,152 (26.33%)	
Large	5,527 (52.89%)	4,691,766 (53.75%)	
Unknown	< 10 cases	< 10 cases	
Location/Teaching Status of Hospital			
Rural	863 (8.25%)	972,637 (11.14%)	< 0.0001
Urban Nonteaching	3,112 (29.78%)	3,727,312 (42.70%)	
Urban Teaching	6,465 (61.87%)	3,998,683 (45.81%)	
Region of Hospital			
Northeast	1,785 (17.08%)	1,553,107 (17.79%)	
Midwest	2,803 (26.82%)	2,360,652 (27.05%)	< 0.0001
South	2,655 (25.41%)	3,092,824 (35.43%)	
West	3,206 (30.68%)	1,721,657 (19.73%)	

*CUD* Cannabis Use Disorder

### Elixhauser comorbidities stratified by CUD

Significant positive associations were found between groups on Elixhauser analysis of comorbidities (Table 3). Patients with CUD were more likely to have comorbid acquired immune deficiency syndrome (AIDS) (0.80% *vs.* 0.05%, *P* < 0.0001), alcohol abuse (20.09% *vs.* 1.09%, *P* < 0.0001), chronic pulmonary disease (23.77% *vs.* 14.64%, *P* < 0.0001), coagulopathy (2.70% *vs.* 2.00%, *P* = 0.0246), depression (19.78% *vs.* 12.17%, *P* < 0.0001), liver disease (5.31% *vs.* 0.94%, *P* < 0.0001), neurological disorders (5.37% *vs.* 3.81%, *P* = 0.0002), obesity (24.65% *vs.* 19.57%, *P* < 0.0001), and psychoses (9.53% *vs.* 1.99%, *P* < 0.0001).

**Table 3:** Elixhauser comorbidities stratified by CUD *vs*. no CUD

	CUD (***n*** = 10,450)	No CUD (***n*** = 8,728,240)	***P***-Value
Acquired Immune Deficiency Syndrome (AIDS)	83 (0.80%)	4,646 (0.05%)	< 0.0001
Alcohol Abuse	2,100 (20.09%)	95,468 (1.09%)	< 0.0001
Deficiency Anemias	1,091 (10.44%)	1,127,581 (12.92%)	0.0017
Rheumatoid Arthritis/Collagen Vascular Disease	447 (4.28%)	335,856 (3.85%)	0.3131
Chronic Blood Loss Anemia	134 (1.28%)	142,540 (16.33%)	0.1989
Congestive Heart Failure	228 (2.18%)	226,512 (2.60%)	0.2620
Chronic Pulmonary Disease	2,484 (23.77%)	1,278,145 (14.64%)	< 0.0001
Coagulopathy	282 (2.70%)	174,526 (2.00%)	0.0246
Depression	2,067 (19.78%)	1,062,505 (12.17%)	< 0.0001
Diabetes (Uncomplicated)	1,349 (12.91%)	1,556,934 (17.84%)	< 0.0001
Diabetes (Complicated)	< 10 cases	137,858 (1.58%)	0.0191
Hypertension	6,025 (57.66%)	5,706,745 (65.38%)	< 0.0001
Hypothyroidism	696 (6.66%)	1,306,443 (14.97%)	< 0.0001
Liver Disease	555 (5.31%)	82,328 (0.94%)	< 0.0001
Lymphoma	< 10 cases	24,127 (0.28%)	0.9284
Fluid and Electrolyte Disorder	921 (8.81%)	740,054 (8.48%)	0.6006
Metastatic Cancer	< 10 cases	12,175 (0.14%)	0.5252
Other Neurological Disorders	561 (5.37%)	332,666 (3.81%)	0.0002
Obesity	2,576 (24.65%)	1,708,050 (19.57%)	< 0.0001
Paralysis	67 (0.64%)	25,084 (0.29%)	0.0022
Peripheral Vascular Disorders	198 (1.89%)	186,509 (2.14%)	0.4478
Psychoses	996 (9.53%)	173,906 (1.99%)	< 0.0001
Pulmonary Circulation Disorders	88 (0.84%)	81,867 (0.94%)	0.6627
Renal Failure	288 (2.75%)	353,326 (4.05%)	0.0023
Solid Tumor without Metastasis	< 10 cases	40,426 (0.46%)	0.6600
Peptic Ulcer Disease Excluding Bleeding	< 10 cases	1,692 (0.02%)	0.6331
Valvular Heart Disease	188 (1.80%)	323,668 (3.71%)	< 0.0001
Weight Loss	60 (0.57%)	29,898 (0.34%)	0.0740

*CUD* Cannabis Use Disorder

Significant negative associations between groups were also observed (Table 3). Patients with CUD were significantly less likely to have comorbid deficiency anemia (10.44% *vs.* 12.92%, *P* = 0.0017), uncomplicated diabetes (12.91% *vs.* 17.84%, *P* < 0.0001), hypertension (57.66% *vs.* 65.38%, *P* < 0.0001), hypothyroidism (6.66% *vs.* 14.97%, *P* < 0.0001), renal failure (2.75% *vs.* 4.05%, *P* = 0.0023), and valvular heart disease (1.80% *vs.* 3.71%, *P* < 0.0001). Table 3 provides a complete description of Elixhauser's comorbidities between the two groups.

### IPTW outcomes, stratified by CUD

No statistically significant differences were found in the composite "any complication" variable between CUD and non-CUD patients (23.45% *vs.* 23.87%, *P* = 0.8149) upon IPTW analysis. Ultimately, 8 of the 9 individual in-hospital complications were not significantly different between groups, with a 79% decrease in odds of developing PE in the CUD cohort as the only significant divergence (OR: 0.21; *P* = 0.0030). CUD was not significantly associated with cardiac complications, GI complications, GU complications, hematoma/seroma, wound dehiscence, postoperative infection, DVT, or postoperative anemia.

There were statistically significant differences in economic and discharge outcomes between cohorts. CUD was associated with a shorter average length of hospital stay (3.04 *vs.* 3.24 days, *P* = 0.0297). Patients with CUD had both significantly higher total ($58,507 *vs.* $50,924, *P* < 0.0001) and daily charges ($22,614 *vs.* $17,955, *P* < 0.0001), compared to patients without CUD. Furthermore, CUD was associated with 45% greater odds of home discharge (OR: 1.45; *P* = 0.0007), and 30% lower odds of rehab discharge (OR: 0.70; *P* = 0.0013). Table 4 provides a complete description of ITPW complications and economic outcomes.

**Table 4:** Complication, economic, and disposition outcomes, stratified by CUD versus no CUD

	CUD	No CUD	OR_**IPTW**_ (95% CI)	***P***-Value
Any Complications	23.45%	23.87%	0.98 (0.81, 1.19)	0.8149
Cardiac Complication	0.63%	0.64%	0.99 (0.55, 1.78)	0.9813
Gastrointestinal (GI) Complication	0.05%	0.27%	0.17 (0.03, 1.16)	0.0706
Genitourinary (GU) Complication	1.11%	0.52%	2.17 (0.66, 7.11)	0.2002
Hematoma/Seroma	0.37%	0.64%	0.58 (0.24, 1.39)	0.2202
Wound Dehiscence	0.21%	0.09%	2.45 (0.80, 7.46)	0.1154
Postoperative Infection	0.12%	0.13%	0.92 (0.23, 3.65)	0.9104
Deep Vein Thrombosis	0.14%	0.33%	0.43 (0.12, 1.53)	0.1923
Pulmonary Embolism	0.07%	0.34%	0.21 (0.08, 0.59)	0.0030
Postoperative Anemia	21.63%	22.08%	0.97 (0.80, 1.19)	0.7926
Home Discharge	73.90%	66.14%	1.45 (1.17, 1.80)	0.0007
Rehab Discharge	25.50%	32.90%	0.70 (0.56, 0.87)	0.0013
Length of Stay (days)	3.04	3.24	--- (---, ---)	0.0297
Total Charges ($)	$58,507	$50,924	--- (---, ---)	< 0.0001

*CUD* Cannabis Use Disorder

## Discussion

As the number of TJA procedures perpetually increases, so does the relative proportion of patients with CUD. This study noted a greater than 5-fold increase in the prevalence of CUD amongst TJA patients over nearly a decade. This trend is similar to the 3-fold increase in CUD for elective surgical admissions that was previously reported between 2006–2015 [2]. Increased CUD prevalence has been attributed to increasing social acceptance and the progressive legalization of cannabis, with the subsequent rise in self-reported cannabis use [1, 2]. This study aimed to characterize this emerging population of TJA patients with CUD by investigating demographic profiles and relevant clinical inpatient postoperative outcomes.

TJA patients with CUD were younger, more often male and non-Hispanic Black, and less likely to be non-Hispanic White. These demographic characteristics mirror cannabis use trends amongst various racial and ethnic groups [22]. Several factors may contribute to CUD within certain demographic groups, including environmental circumstances, community norms, increased blunt usage, and socioeconomic barriers to healthcare [22]. Additionally, while overall CUD prevalence is higher among men, women with CUD face unique challenges, including higher rates of comorbid depression, anxiety, and more severe withdrawal symptoms [22–25]. It is important for clinicians to consider these factors in the immediate postoperative in-hospital setting, where limited access to cannabis may incite withdrawal symptoms. When addressing substance use in the perioperative setting, careful attention should be directed to the unique barriers, challenges, and characteristics faced by these patients.

Patients with CUD had a mean age of 55.35 years, against 66.21 years in the control group. This finding helps explain the significant differences in expected primary payor, as patients with CUD had higher rates of Medicaid (coverage for low-income individuals of all ages) and lower rates of Medicare (coverage for adults ≥ 65 years and those with disabilities) insurance compared to the control group. It has been previously shown that most adults with CUD have a relatively lower income, and therefore may qualify for Medicaid benefits [26]. Thus, as the majority of funding for substance use treatment comes from public sources, it is critical for public health initiatives to expand coverage and resources available for lower-income individuals and improving the infrastructure for screening, intervention, and treatment for substance use disorders [26, 27].

Several significant comorbidity associations were found in the CUD cohort in this study. CUD patients had significantly higher rates of comorbidities that are associated with risky behaviors and disinhibition, such as acquired immune deficiency syndrome (AIDS), alcohol abuse, and liver pathology [28–31]. This supports previous literature, showing marijuana use and other substance abuse disorders are associated with inhibitory control impairment and subsequent risky behaviors [29–31]. Patients with CUD also had significantly higher rates of comorbid mental illness. The high degree of comorbid depression and psychoses in cannabis users has already been well documented [32–34]. Although these studies have not established causality between CUD and psychiatric disorders, there is sufficient evidence to justify interventions for harm reduction and prevention among at-risk populations [33, 34]. This association is especially important in the arthroplasty perioperative setting, as mental illness is highly associated with increased morbidity following total joint arthroplasty [35, 36]. As legislation has evolved over recent years, attitudes have shifted towards increased acceptability and views that cannabis is a harmless substance [37]. However, the associated comorbidities noted in CUD patients are independent risk factors that may predispose patients to poor health outcomes following TJA [38–40]. With the rise in the prevalence of cannabis use and dependence, it is increasingly important for clinicians to account for these comorbidities in the perioperative setting.

This study found no significant differences in the overall complication rates between groups. The only significant difference upon scrutinizing individual complications involved a lower rate of PE in CUD patients. Interestingly, there was no difference in DVT rate between groups. This contradicted previous literature correlating CUD with higher rates of thromboembolic complications caused by changes in hematological parameters and platelet morphology with cannabis use [41–44]. While statistical methodological differences may play a role in this observation, this paradoxical finding may also potentially be explained by aggressive perioperative management and monitoring of patients with CUD for PE, ultimately leading to increased prevention. Regardless, more investigation into this relationship is warranted. For all other immediate postoperative complications, isolating CUD through propensity-weighted analysis showed no significant difference between cohorts. This is an encouraging supplement to recent literature showing no differences in perioperative clinical outcomes between similar cohorts undergoing major elective surgeries and arthroplasty [2, 3].

Analysis of economic outcomes revealed that CUD patients had significantly shorter length of stay (LOS) and higher rates of home discharge following primary TJA compared to the control group. This contrasted with previous reports of increased LOS for patients with CUD following primary TJA [13, 45]. Differences in statistical methodology likely account for these differences, as this study's propensity weighting methodology allowed for controlling a large number of potentially confounding factors for LOS and discharge disposition. It is intuitive that patients with CUD, who are, by definition, dependent on cannabis or have a problematic pattern of cannabis use, would be incentivized to leave the hospital as soon as possible and return home to continue use of cannabis and potentially other substances. Because such use may be associated with problematic behavioral changes and abandonment of social, occupational, or recreational activities, these patients may be at risk for worse postoperative and overall health outcomes in the postoperative, post-discharge period. In contrast, the preoperative and in-hospital period, during which a multidisciplinary team has full access to care for these patients, can thus serve as an opportune time for comprehensive social and medical intervention. As such, orthopedic surgeons and the multidisciplinary medical and social service team should remain aware of the risks these patients face, and perioperative interventions should be considered to optimize both long-term outcomes and general health improvement in these patients.

This study is limited for several reasons. A majority of these limitations are inherently related to study design. A large national registry database such as the NIS provides a rich source of data to analyze, however, input of these data is often inconsistent and/or incomplete [46]. Additional drawbacks stemmed from inherent limitations in the NIS database [47]. The database only reports on current hospital admissions without data on longer-term follow-up. Therefore, postoperative outcome measures only related to the immediate in-hospital setting for this study. For example, this database allows for analysis of length of stay and discharge disposition, but excludes specific data on timing for return to work or rehabilitation requirements. Literature would benefit from future studies focusing on long-term outcomes of CUD and TJA building on the epidemiological and demographic characteristics of patients with CUD at the time of primary TJA presented here. The NIS database is also limited as it reports data in aggregate, and individual patient granularity is not profiled. Findings in this study showed significant differences in "total charges" of admission between CUD and non-CUD study groups. While the NIS database keeps a record of "total charges" accrued during a patient's hospital admission, these are not itemized into individual charges and thus prevents any closer analysis of variations in hospital charges between patients. Similarly, specific postoperative pain medication requirements for individual patients are not recorded in the NIS database. Further studies on this topic would benefit from including a more thorough analysis of the patients' postoperative course in regard to pain medication requirements. Lastly, this study only included patients with CUD, a subset which represents only about 10% of the 193 million worldwide cannabis users [48]. Future studies may evaluate how the characteristics and postoperative outcomes of TJA patients with classified CUD compare to those self-reporting recreational cannabis use. Unfortunately, this distinction cannot be made within the current NIS database.

This study has numerous strengths despite the unavoidable limitations. This study, to the authors' knowledge, constituted the largest investigation of epidemiological and demographic characteristics of patients with CUD undergoing primary TJA. The length and size of this study allowed for a robust understanding of demographic profiles for the growing population of patients with CUD undergoing primary TJA. Additionally, information on clinical and economic postoperative outcomes for these patients provides a better understanding of the implications of cannabis use in the immediate postoperative setting. While cannabis use is becoming increasingly legalized throughout the United States, CUD is also a relevant problem in other countries around the world. While high-income countries maintain the highest prevalence of cannabis use, there is a growing prevalence of cannabis use in low-income and middle-income countries [48]. Specifically, there is a higher estimated use in North Africa (12.4%), West and Central Africa (12.4%) and Oceania (10.3%) compared to Asia (1.8%), North Africa (4.3%) and Eastern and Southern Europe (2.4%) [48]. This study's findings can provide physicians worldwide with a better understanding of CUD and serve this growing patient population.

In conclusion, this study found a significant and greater-than-five-fold increase in the prevalence of CUD among patients undergoing primary TJA from 2006–2015. Progressive legalization of cannabis use makes it increasingly important for clinicians to understand the characteristics of this evolving patient population. As this growing population continues to evolve, understanding their comorbidities, behavioral characteristics, and postoperative clinical and economic outcomes allow orthopedic surgeons and the multidisciplinary healthcare teams to better tailor their care and management of these patients. Further research should aim to more closely and comparatively assess the demographic profile of patients with both recreational use and substance use disorder, along with potential barriers in their access to medical care. This understanding should be associated with the expansion and improvement of public health initiatives and the development of frameworks to better deliver substance use screenings and interventions to this patient population. Such initiatives, combined with the development of standardized perioperative protocols, have the potential to optimize postsurgical and overall health outcomes in this at-risk patient population.

## Data Availability

The data that support the findings of this study are available from the corresponding author upon reasonable request.

## Abbreviations

THA: Total hip arthroplasty
TKA: Total knee arthroplasty
TJA: Total joint arthroplasty
CUD: Cannabis use disorder
LOS: Length of stay
NIS: National Inpatient Sample

## References

[1] Jennings JM, Williams MA, Levy DL, Johnson RM, Eschen CL, Dennis DA. Has self-reported marijuana use changed in patients undergoing total joint arthroplasty after the legalization of marijuana? Clin Orthop Relat Res. 2019;477(1):95–100. 10.1097/CORR.0000000000000339. 30794232 10.1097/CORR.0000000000000339 PMC6345315

[2] Goel A, McGuinness B, Jivraj NK, Wijeysundera DN, Mittleman MA, Bateman BT, et al. Cannabis use disorder and perioperative outcomes in major elective surgeries: a retrospective cohort analysis. Anesthesiology. 2020;132(4):625–35. 10.1097/aln.0000000000003067. 31789638 10.1097/ALN.0000000000003067

[3] Jennings JM, Angerame MR, Eschen CL, Phocas AJ, Dennis DA. Cannabis use does not affect outcomes after total knee arthroplasty. J Arthroplast. 2019;34(8):1667–9. 10.1016/j.arth.2019.04.015. 10.1016/j.arth.2019.04.015 31072746

[4] Bicket MC, McGinty EE. Cannabis use disorder and surgery: a budding problem? Anesthesiology. 2020;132(4):612–3. 10.1097/aln.0000000000003135. 32053558 10.1097/ALN.0000000000003135 PMC7071976

[5] Miller G. Pot and pain. Science. 2016;354(6312):566–8. 10.1126/science.354.6312.566. 27811265 10.1126/science.354.6312.566

[6] Liu CW, Bhatia A, Buzon-Tan A, Walker S, Ilangomaran D, Kara J, et al. Weeding out the problem: the impact of preoperative cannabinoid use on pain in the perioperative period. Anesth Analg. 2019;129(3):874–81. 10.1213/ANE.0000000000003963. 31425232 10.1213/ANE.0000000000003963

[7] Agarwalla A, Liu JN, Gowd AK, Amin NH, Werner BC. Differential use of narcotics in total hip arthroplasty: a comparative matched analysis between osteoarthritis and femoral neck fracture. J Arthroplast. 2020;35(2):471–6. 10.1016/j.arth.2019.09.004. 10.1016/j.arth.2019.09.004 31564525

[8] Kim P, Yamashita T, Shen JJ, Park SM, Chun SY, Kim SJ, et al. Dissociation between the growing opioid demands and drug policy directions among the U.S. older adults with degenerative joint diseases. Med (United States). 2019;98(28). 10.1097/MD.0000000000016169. 10.1097/MD.0000000000016169 PMC6641693 31305399

[9] Anciano Granadillo V, Cancienne JM, Gwathmey FW, Werner BC. Perioperative opioid analgesics and hip arthroscopy: trends, risk factors for prolonged use, and complications. Arthrosc - J Arthrosc Relat Surg. 2018;34(8):2359–67. 10.1016/j.arthro.2018.03.016. 10.1016/j.arthro.2018.03.016 29730217

[10] Law TY, Kurowicki J, Rosas S, Sabeh K, Summers S, Hubbard Z, et al. Cannabis use increases risk for revision after total knee arthroplasty. J Long-Term Eff Med Implants. 2018;28(2):125–30. 10.1615/JLongTermEffMedImplants.2018027401. 30317962 10.1615/JLongTermEffMedImplants.2018027401 PMC6396273

[11] Best MJ, Buller LT, Klika AK, Barsoum WK. Outcomes following primary total hip or knee arthroplasty in substance misusers. J Arthroplast. 2015;30(7):1137–41. 10.1016/j.arth.2015.01.052. 10.1016/j.arth.2015.01.052 25765129

[12] Roche M, Law TY, Sodhi N, Rosas S, Kurowicki J, Disla S, et al. Incidence of drug abuse in revision total knee arthroplasty population. J Knee Surg. 2018;31(10):928–33. 10.1055/s-0038-1669915. 30193389 10.1055/s-0038-1669915 PMC6427918

[13] Vakharia RM, Mannino A, Salem HS, Roche MW, Wong CHJ, Mont MA. The association between cannabis use disorder and the outcome following primary total hip arthroplasty: analysis of a nationwide administrative claims database. Bone Jt J. 2021;103-B:111–5. 10.1302/0301-620X.103B7.BJJ-2020-2424.R1. 10.1302/0301-620X.103B7.BJJ-2020-2424.R1 34192906

[14] Cerdá M, Mauro C, Hamilton A, et al. Association between recreational marijuana legalization in the United States and changes in marijuana use and cannabis use disorder from 2008 to 2016. JAMA Psychiatry. 2020;77(2):165–71. 10.1001/jamapsychiatry.2019.3254. 31722000 10.1001/jamapsychiatry.2019.3254 PMC6865220

[15] Kurtz S, Ong K, Lau E, Mowat F, Halpern M. Projections of primary and revision hip and knee Arthroplasty in the United States from 2005 to 2030. J Bone Jt Surg. 2007;89(4):780. 10.2106/JBJS.F.00222. 10.2106/JBJS.F.00222 17403800

[16] Trend Weights for HCUP NIS Data. Rockville: Agency for Healthcare Research and Quality; https://www.hcup-us.ahrq.gov/db/nation/nis/trendwghts.jsp. Accessed 17 Nov 2022.

[17] Houchens R, Elixhauser A. (2012) Final Report on Calculating Nationwide Inpatient Sample (NIS) Variances for Data Years 2011 and Earlier. U.S. Agency for Healthcare Research and Quality.

[18] Houchens R, Ross D, Elixhauser A. (2015) Final Report on Calculating National Inpatient Sample (NIS) Variances for Data Years 2012 and Later. http://www.hcup-us.ahrq.gov/reports/methods/methods.jsp. HCUPNIS.

[19] Dugoff EH, Schuler M, Stuart EA. Generalizing observational study results: applying propensity score methods to complex surveys. Health Serv Res. 2014;49(1):284–303. 10.1111/1475-6773.12090. 23855598 10.1111/1475-6773.12090 PMC3894255

[20] Stuart EA. Matching methods for causal inference: a review and a look forward. Stat Sci. 2010;25(1):1–21. 20871802 10.1214/09-STS313 PMC2943670

[21] Ondeck NT, Bohl DD, Bovonratwet P, McLynn RP, Cui JJ, Grauer JN. Discriminative ability of Elixhauser’s comorbidity measure is superior to other comorbidity scores for inpatient adverse outcomes after total hip arthroplasty. J Arthroplast. 2018;33(1):250–7. 10.1016/j.arth.2017.08.032. 10.1016/j.arth.2017.08.032 28927567

[22] Wu LT, Zhu H, Swartz MS. Trends in cannabis use disorders among racial/ethnic population groups in the United States. Drug Alcohol Depend. 2016;165:181–90. 10.1016/j.drugalcdep.2016.06.002. 27317045 10.1016/j.drugalcdep.2016.06.002 PMC4939114

[23] Compton WM, Grant BF, Colliver JD, Glantz MD, Stinson FS. Prevalence of marijuana use disorders in the United States: 1991-1992 and 2001-2002. J Am Med Assoc. 2004;291(17):2114–21. 10.1001/jama.291.17.2114. 10.1001/jama.291.17.2114 15126440

[24] Herrmann ES, Weerts EM, Vandrey R. Sex differences in cannabis withdrawal symptoms among treatment-seeking cannabis users. Exp Clin Psychopharmacol. 2015;23(6):415–21. 10.1037/pha0000053. 26461168 10.1037/pha0000053 PMC4747417

[25] Khan SS, Secades-Villa R, Okuda M, et al. Gender differences in cannabis use disorders: results from the National Epidemiologic Survey of alcohol and related conditions. Drug Alcohol Depend. 2013;130(1-3):101–8. 10.1016/j.drugalcdep.2012.10.015. 23182839 10.1016/j.drugalcdep.2012.10.015 PMC3586748

[26] Wu LT, Zhu H, Mannelli P, Swartz MS. Prevalence and correlates of treatment utilization among adults with cannabis use disorder in the United States. Drug Alcohol Depend. 2017;177:153–62. 10.1016/j.drugalcdep.2017.03.037.Prevalence. 28599214 10.1016/j.drugalcdep.2017.03.037 PMC5538354

[27] Mark TL, Levit KR, Vandivort-Warren R, Buck JA, Coffey RM. Changes in US spending on mental health and substance abuse treatment, 1986-2005, and implications for policy. Health Aff. 2011;30(2):284–92. 10.1377/hlthaff.2010.0765. 10.1377/hlthaff.2010.0765 21289350

[28] Du P, Crook T, Whitener C, Albright P, Greenawalt D, Zurlo J. HIV transmission risk behaviors among people living with HIV/ AIDS: the need to integrate HIV prevention interventions and public health strategies into HIV care ping. J Public Health Manag Pract. 2015;21(2):1–10. 24335609 10.1097/PHH.0000000000000038 PMC4051857

[29] Smith JL, Mattick RP, Jamadar SD, Iredale JM. Deficits in behavioural inhibition in substance abuse and addiction: a meta-analysis. Drug Alcohol Depend. 2014;145:1–33. 10.1016/j.drugalcdep.2014.08.009. 25195081 10.1016/j.drugalcdep.2014.08.009

[30] Andrade LF, Carroll KM, Petry NM. Marijuana use is associated with risky sexual behaviors in treatment-seeking polysubstance abusers. Am J Drug Alcohol Abuse. 2013;39(4):266–71. 10.3109/00952990.2013.803112. 23841867 10.3109/00952990.2013.803112 PMC3793248

[31] Alarcó-Rosales R, Sánchez-SanSegundo M, Ferrer-Cascales R, Albaladejo-Blázquez N, Ruiz-Robledillo N, Delvecchio E, et al. Relationships between problematic cannabis use and risky behaviors in Spanish adolescents. Int J Environ Res Public Health. 2019;16(17). 10.3390/ijerph16173029. 10.3390/ijerph16173029 PMC6747259 31438581

[32] Lev-Ran S, Roerecke M, Le Foll B, George TP, McKenzie K, Rehm J. The association between cannabis use and depression: a systematic review and meta-analysis of longitudinal studies. Psychol Med. 2014;44(4):797–810. 10.1017/S0033291713001438. 23795762 10.1017/S0033291713001438

[33] Marconi A, Di Forti M, Lewis CM, Murray RM, Vassos E. Meta-analysis of the association between the level of cannabis use and risk of psychosis. Schizophr Bull. 2016;42(5):1262–9. 10.1093/schbul/sbw003. 26884547 10.1093/schbul/sbw003 PMC4988731

[34] Onaemo VN, Fawehinmi TO, D’Arcy C. Comorbid cannabis use disorder with major depression and generalized anxiety disorder: a systematic review with meta-analysis of nationally representative epidemiological surveys. J Affect Disord. 2021;281:467–75. 10.1016/j.jad.2020.12.043. 33360749 10.1016/j.jad.2020.12.043

[35] Zalikha L, Karabon PMs, Hajj Hussein IP, El-Othmani MMM. Anxiety and depression impact on Inhospital complications and outcomes after total knee and hip arthroplasty: a propensity score-weighted retrospective analysis. J Am Acad Orthop Surg. 2021;29(20):873–84. 10.5435/JAAOS-D-20-00721. 10.5435/JAAOS-D-20-00721 34525481

[36] Kamalapathy P, Kurker KP, Althoff AD, Browne JA, Werner BC. The impact of mental illness on postoperative adverse outcomes after outpatient joint surgery. J Arthroplast. 2021;36(8):2734–41. 10.1016/j.arth.2021.04.002. 10.1016/j.arth.2021.04.002 33896669

[37] Hasin DS. US epidemiology of cannabis use and associated problems. Neuropsychopharmacology. 2018;43(1):195–212. 10.1038/npp.2017.198. 28853439 10.1038/npp.2017.198 PMC5719106

[38] O’Neill SC, Queally JM, Hickey A, Mulhall KJ. Outcome of total hip and knee arthroplasty in HIV-infected patients: a systematic review. Orthop Rev (Pavia). 2019;11(1):24–30. 10.4081/or.2019.8020. 10.4081/or.2019.8020 PMC6452098 30996842

[39] Best MJ, Buller LT, Gosthe RG, Klika AK, Barsoum WK. Alcohol misuse is an independent risk factor for poorer postoperative outcomes following primary total hip and total knee arthroplasty. J Arthroplast. 2015;30(8):1293–8. 10.1016/j.arth.2015.02.028. 10.1016/j.arth.2015.02.028 25769745

[40] Gold PA, Garbarino LJ, Anis HK, et al. The cumulative effect of substance abuse disorders and depression on postoperative complications after primary total knee arthroplasty. J Arthroplast. 2020;35(6):151–7. 10.1016/j.arth.2020.01.027. 10.1016/j.arth.2020.01.027 32061474

[41] Vakharia RM, Sodhi N, Anis HK, Ehiorobo JO, Mont MA, Roche MW. Patients who have cannabis use disorder have higher rates of venous Thromboemboli, readmission rates, and costs following primary Total knee Arthroplasty. J Arthroplast. 2020;35(4):997–1002. 10.1016/j.arth.2019.11.035. 10.1016/j.arth.2019.11.035 31973970

[42] Guzel D, Yazici AB, Yazici E, Erol A. Alterations of the hematologic cells in synthetic cannabinoid users. J Clin Lab Anal. 2017;31(6):1–7. 10.1002/jcla.22131. 10.1002/jcla.22131 PMC6817267 28169460

[43] Deusch E, Kress HG, Kraft B, Kozek-Langenecker SA. The procoagulatory effects of delta-9-tetrahydrocannabinol in human platelets. Anesth Analg. 2004;99(4):1127–30. 10.1213/01.ANE.0000131505.03006.74. 15385362 10.1213/01.ANE.0000131505.03006.74

[44] Baldassarri S, Bertoni A, Bagarotti A, Sarasso C, Zanfa M, Catani MV, et al. The endocannabinoid 2-arachidonoylglycerol activates human platelets through non-CB1/CB2 receptors. J Thromb Haemost. 2008;6(10):1772–9. 10.1111/j.1538-7836.2008.03093.x. 18647220 10.1111/j.1538-7836.2008.03093.x

[45] Runner RP, Luu AN, Nassif NA, Scudday TA, Patel JJ, Barnett SL, et al. Use of Tetrahydrocannabinol and Cannabidiol products in the perioperative period around primary unilateral total hip and knee arthroplasty. J Arthroplast. 2020. 10.1016/j.arth.2020.01.077. 10.1016/j.arth.2020.01.077 32173619

[46] Pass HI. Medical registries: continued attempts for robust quality data. J Thorac Oncol. 2010;5(6 SUPPL. 2):198–9. 10.1097/JTO.0b013e3181dcf957. 10.1097/JTO.0b013e3181dcf957 20502263

[47] Bozic KJ, Bashyal RK, Anthony SG, Chiu V, Shulman B, Rubash HE. Is administratively coded comorbidity and complication data in total joint arthroplasty valid? Clin Orthop Relat Res. 2013;471:201–5. 22528384 10.1007/s11999-012-2352-1 PMC3528892

[48] Connor J, Stjepanović D, Le Foll B, Hoch E, Budney A, Hall W. Cannabis use and cannabis use disorder. Nat Rev Dis Prim. 2021;7(1). 10.1038/s41572-021-00247-4. 10.1038/s41572-021-00247-4 PMC8655458 33627670

